# Facile preparation of nickel/carbonized wood nanocomposite for environmentally friendly supercapacitor electrodes

**DOI:** 10.1038/srep33659

**Published:** 2016-09-21

**Authors:** Haritha Sree Yaddanapudi, Kun Tian, Shiang Teng, Ashutosh Tiwari

**Affiliations:** 1 Nanostructured Materials Research Laboratory, Department of Materials Science and Engineering, University of Utah, Salt Lake City, Utah, 84112, USA.

## Abstract

We are reporting a facile way to prepare nickel/carbon nanocomposites from wood as a novel electrode material for supercapacitors. The surface morphology and the structure of the as-prepared electrodes were studied by using X-ray diffraction (XRD), scanning electron microscopy (SEM), transmission electron microscopy (TEM) and X-ray photoelectron spectroscopy (XPS). The results indicate that after high-temperature carbonization process, the wood is converted into graphitic carbon with nickel nanoparticles uniformly distributed within the three dimensional structure of the wood. Electrochemical characterization such as cyclic voltammetry (CV), electrochemical impedance spectroscopy (EIS) and galvanostatic charge-discharge measurements were conducted. These results showed that the introduction of nickel into the carbonized wood improves the specific capacitance and the cyclic stability of the nanocomposite electrode over that of the pure carbonized wood electrode. The composite electrode displayed an enhanced capacitive performance of 3616 F/g at 8 A/g, and showed an excellent capacitance retention after 6000 charge-discharge cycles. These results endow the nickel nanoparticles impregnated carbonized wood with a great potential for future application in supercapacitors.

Due to growing consumption of electricity and concerns about pollution and global warming, there has been a thrust to move towards the “green” methods of power generation. With this move to green power, it has become important to develop energy storage devices which are equally environmentally friendly and at the same time are cost effective and have good storage capacity^1^,^2^,^3^. Amongst the various energy storage devices, supercapacitors have gained a lot of attention recently due to their high energy density, excellent power density, long cyclic lifetime and an extended working temperature range^4^,^5^,^6^,^7^.

Based on their charge storage mechanism, supercapacitors are classified into two classes (a) electrical double layer capacitors (EDLC) and (b) pseudocapacitors^8^. Most EDLCs utilize carbon based materials as electrodes. These include, but are not limited to, carbon nanofibers, carbon nanotubes, activated carbon, and carbon aerogels. These materials have good thermal and chemical stability, high surface area, and are easily polarizable. Furthermore, they are of low cost and simple processability^9^. However, performance of EDLC systems is limited due to their low energy density, therefore recently several investigations have focused on pseudocapacitors which utilize the charge storage by faradic reactions. Metal and metal oxide materials are commonly used as electrodes in pseudocapacitors^10^,^11^,^12^,^13^,^14^,^15^,^16^.

Although metal/oxide based electrodes offer many benefits when compared to carbon based materials, they also have a drawback of small operating voltage window. To overcome this drawback, supercapacitors utilizing the composites of carbon and metal/oxides have been proposed which combine the mechanisms of both double-layer capacitance as well as pseudocapacitance to increase the overall capacitance of the system^17^. These supercapacitors not only exhibit higher energy and power densities (compared to EDLCs or pseudocapacitors alone), but also exhibit a much higher rate capability. Some of the composite materials which have been explored recently include MnO_2_/carbon nanotubes (CNTs), NiO/CNTs, and Ni(OH)_2_/CNTs^18^,^19^,^20^.

In an earlier study, Teng *et al*. showed that because of its porous structure and high effective surface area, carbonized wood can be used as a supercapacitor electrode^21^. In the present study, we are showing that the electrochemical performance of the wood based supercapacitor electrodes can be enhanced tremendously by the addition of nickel nanoparticles (NiNPs). An environmentally friendly method of fabricating nickel/carbonized wood nanocomposite electrodes for efficient supercapacitor application is being reported herewith.

## Methods

### Chemicals

Ammonium hydroxide (EMD Millipore, USA, 28% to 30% Assay), nickel nitrate hexahydrate (NiNO_3_∙6H_2_O, Alfa Aesar, UK, 98%), and potassium hydroxide (KOH, Fischer Scientific, USA, >85%) analytical reagents were used as received without any further purification. Deionized water was used as the solvent in all the experiments performed.

### Preparation of NiNPs impregnated carbonized wood electrodes

Firstly, the beech wood was cut into disc shaped samples and rinsed under running water to remove the sawdust. The washed wood pieces were then boiled in 1 M ammonia solution at 90 °C for 5 hours. This helped in removing the resins and additional impurities present in the wood. The skin of the boiled wood samples was later removed and the wood pieces were then transferred into a box furnace for dehydration at a temperature of 120 °C for 3 hours. Later, the dehydrated wood samples were immersed in NiNO_3_ solution for 4 hours at 90 °C. The concentration of the NiNO_3_ solution was varied from 0.5 M to 3 M and the samples were designated as Ni 0.5 M, Ni 1 M, Ni 2 M, and Ni 3 M. The immersion of wood in the NiNO_3_ solution impregnated the nickel ions into the pores of the dehydrated wood sample. The NiNO_3_ impregnated wood samples were then transferred into a tube furnace for carbonization at 900 °C under N_2_ atmosphere for 1 hour. The above high temperature process also resulted in the formation of NiNPs because of the reduction of NiNO_3_. The gravimetric concentration of Ni present in the final electrode was estimated to be 5.4%, 14.8%, 20.4%, and 27.5% for Ni 0.5 M, Ni 1 M, Ni 2 M and Ni 3 M, respectively (See Fig. S1 in the supporting information for more details). The resulting NiNPs impregnated carbonized wood samples were used for further material characterization and electrochemical testing.

Here, it is important to note that in most of the studies reported in literature the electrode comprises of a layer of active material coated on a metallic substrate. This metallic substrate acts as a current collector. However, in our case both the active material as well as the supporting electrode material are made of same piece of wood. In order to estimate the amount of active material that was participating in electrochemical reaction, we performed XPS studies on the charged electrode with successive mechanical etching. [See Fig. S2 in the supporting information section for more detail]. By monitoring the peak position of Ni 2p_1/2_ and Ni 2p_3/2_ at different depths of the charged sample, ionic states of nickel were determined. It was found that about 0.2 mm of the outer surface of the electrode was actually participating in the reaction. The mass of this layer is used for calculating the specific capacitance.

### Characterizations

X-Ray powder diffractograms were recorded using a Philips X’Pert X-Ray Diffractometer at a scan rate of 0.05° s^−1^ with 2θ ranging from 10° to 90° using Cu K_α_ radiation. The surface morphology of the prepared samples was characterized by using an FEI Quanta 600 FEG scanning electron microscope. To further obtain the information on microstructure and chemical composition, a transmission electron microscope (JEOL JEM-2800) equipped with an energy-dispersive x-ray spectroscope (EDX) was used. X-ray photoelectron spectroscopy (XPS) measurements were performed using Kratos Axis Ultra DLD to determine the ionic states of nickel present in the electrode. The electrochemical measurements were carried out using a Reference 600^TM^ from Gamry Instruments with CV, EIS, and galvanostatic charge discharge functions. These measurements were performed with a three-electrode electrochemical cell where the prepared NiNPs impregnated carbonized wood served as the working electrode, platinum sheet as the counter electrode, and Ag/AgCl as the reference electrode. 5 M KOH solution was used as the electrolyte. The CV measurements were carried out at sweep rates over a range of 1 mV/s to 50 mV/s. EIS measurements were performed at a frequency range from 0.1 Hz to 10^6^ Hz with an AC voltage of 0.1 V. The galvanostatic charge-discharge cycling was performed over a potential range from 0 V to 0.5 V. To investigate the life time of the supercapacitors, cyclic life tests for 6000 cycles of repeated charge-discharge experiments were conducted.

## Results and Discussion

### Structural Characterization

**Figure 1:**
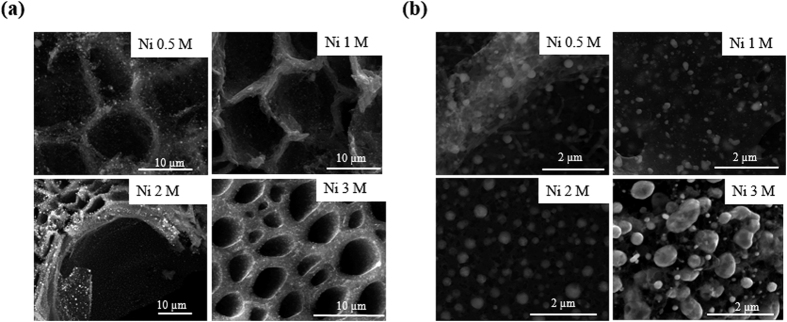
SEM images of NiNPs impregnated carbonized wood electrodes at (**a**) Low magnification, (**b**) high magnification.

Figure 1 shows the SEM images of various NiNPs impregnated carbonized wood electrodes at low magnification (a) and high magnification (b). The low magnification images show the interconnected channels for all the samples, indicating the retention of the three dimensional structure of the wood even after the carbonization at 900 °C. The high magnification SEM images indicate that the NiNPs are uniformly distributed in pores across and deep within the sample surface. It is observed that the size of NiNPs steadily increases with the increase in the NiNO_3_ concentration. From the SEM images, the average sizes of NiNPs for Ni 0.5 M, Ni 1 M, Ni 2 M and Ni 3 M were measured and found to be around 170 nm, 210 nm, 300 nm and 475 nm, respectively.

**Figure 2:**
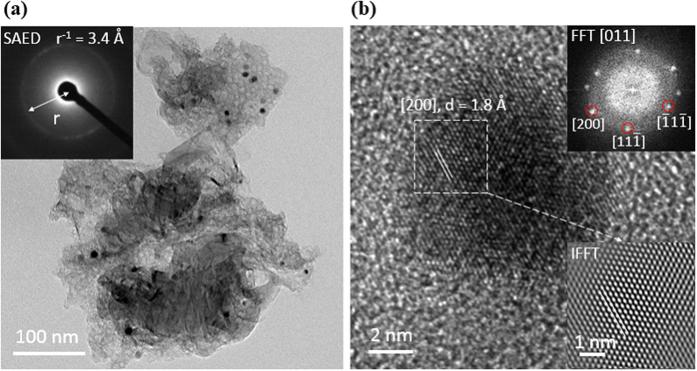
TEM images of NiNPs impregnated carbonized wood electrodes at: (**a**) Low magnification image; Inset shows the SAED pattern, (**b**) high magnification image of NiNP; Inset shows the FFT of NiNP and IFFT of the enclosure region in (**b**).

**Figure 3:**
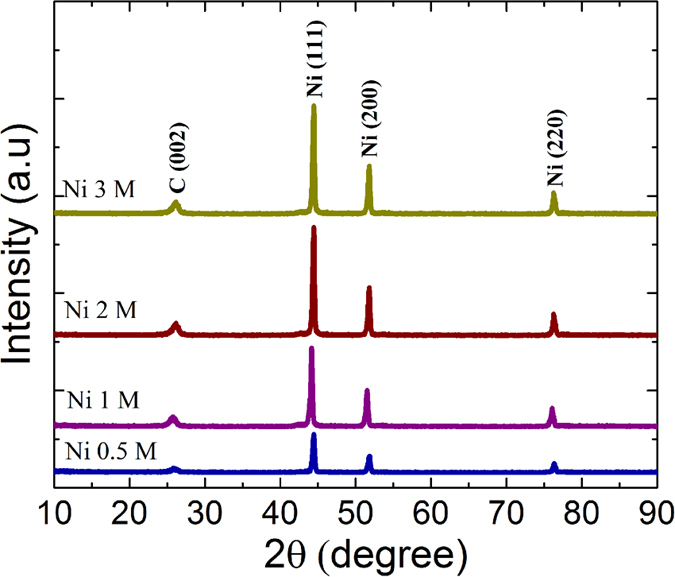
XRD patterns of Ni 0.5 M, Ni 1 M, Ni 2 M and Ni 3 M.

Further to reveal the microstructure of NiNPs, TEM imaging on NiNPs impregnated carbonized wood was performed. Figure 2(a) shows a low magnification TEM image of NiNPs impregnated carbonized wood (for Ni 2M sample). An obvious contrast in the image comprising of dark spots gives a clear indication of presence of small nanoparticles. Moreover, the selected-area electron diffraction (SAED) pattern shown in the inset of Fig. 2(a) indicates a well-defined diffraction ring pattern which can be indexed to the (002) plane of graphite thus suggesting a highly conductive nature of NiNPs impregnated carbonized wood electrodes. A high resolution transmission electron microscopy (HRTEM) image of NiNP is shown in Fig. 2(b). The HRTEM image shows well-resolved lattice fringes with an equal interplanar distance of 0.18 nm that corresponds to the d-spacing of (200) plane of cubic phase nickel, revealing a high crystallization feature of NiNPs. A fast Fourier transformation (FFT) pattern (top inset of Fig. 2b) is further collected from the area of the lattice fringes which exhibits a zone axis of [011] for NiNPs. The lattice planes of the FFT are indexed as (200), (11-1), and (-11-1) respectively which are consistent with the XRD results (shown in Fig. 3). The bottom inset of Fig. 2(b) demonstrates the inverse fast Fourier transformation (IFFT) taken from the enclosure region represented in the HRTEM image. Additionally, we also performed dark-field scanning transmission electron microscopy (DF-STEM), bright-field scanning transmission electron microscopy (BF-STEM) and EDX mapping to study the elemental distribution in NiNPs impregnated carbonized wood electrodes. Results for Ni 2M sample are shown in Fig. S3 in the supporting information section. The DF-STEM and BF-STEM images further reveal a clear formation of nanoparticles in the electrode. The EDX-STEM elemental mapping images shown in Fig. S3 correspond to the elements of C, Ni collected over the electrode region. To further determine the surface composition and electronic state of nickel, XPS analysis is carried out on the as prepared electrode (See Fig. S2 of supporting information section for more information). Nickel was detected with its corresponding electronic state to be 0 implying the presence of metallic nickel in the as prepared electrode.

X-ray diffraction patterns recorded from the NiNPs impregnated carbonized wood electrodes are shown in Fig. 3. As can be seen, the observed XRD peaks indicate the presence of pure cubic nickel phase (JCPDS Reference Number 00-004-0850) and hexagonal graphite (JCPDS Reference Number 00-056-0159) phase. No other phases were detected.

### Cyclic Voltammetry Measurements

**Figure 4:**
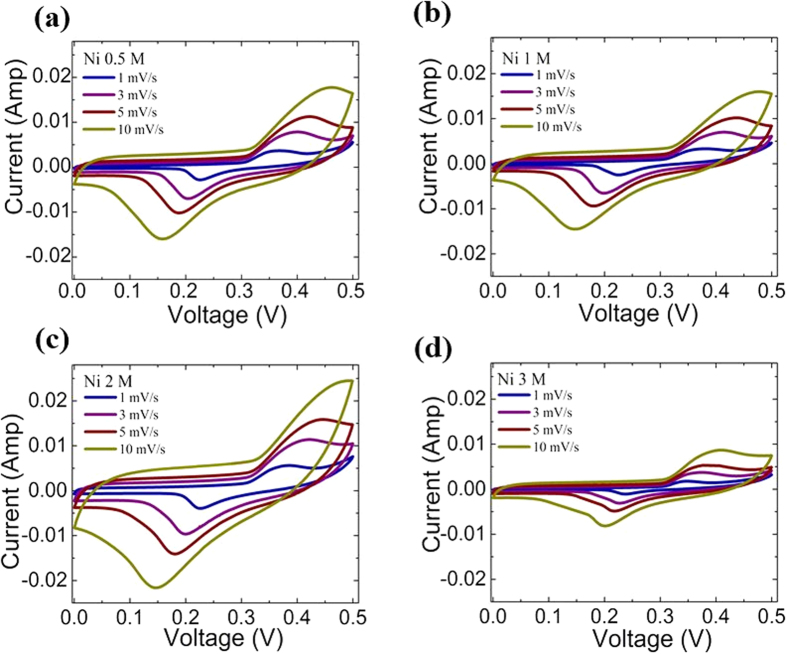
CV curves of Ni 0.5 M, Ni 1 M, Ni 2 M and Ni 3 M at different scan rates of 1 mV/s, 3 mV/s, 5 mV/s and 10 mV/s.

The CV measurements on the NiNPs impregnated carbonized wood were performed at sweep rates varying from 1 mV/s to 50 mV/s and over a potential range of 0 V–0.5 V (vs. Ag/AgCl). Results for sweep rates of 1 mV/s, 3 mV/s, 5 mV/s and 10 mV/s are shown in Fig. 4 and for higher sweep rates of 25 mV/s, 30 mV/s, 40 mV/s and 50 mV/s are shown in Fig. S4 of the supporting information section. For comparison, CV measurements on pure carbonized wood were also performed under identical conditions. Results are shown in Fig. S5 of the supporting information section. It can be seen that the CV curves for all the NiNPs impregnated carbonized wood samples show peaks which are indicative of the redox reactions and suggest the psuedocapacitive behavior of the electrodes. The two characteristic peaks, i.e. the anodic peak at the positive current and the cathodic peak at the negative current, are due to the oxidation of nickel to highly hydrated α-Ni(OH)_2_ (charging) and the reduction of α-Ni(OH)_2_ to nickel (discharging), respectively. The charging process follows the forward direction of the following redox reaction^22^,^23^,^24^,^25^: Ni + 2OH^−^ ↔ α-Ni (OH)_2_ + 2e^−^ while the discharging process follows the backward reaction. With the increase in the scan rate, both the anodic and cathodic peak currents increase (See Fig. S6 of the supporting information). Additionally, there is an increase in the separation distance between the anodic peak potential and the cathodic peak potential which indicate a diffusion-controlled quasi-reversible charge transfer process of the ions^26^ (See Fig. S7 of the supporting information).

The integral area of the CV curve over scan rate is directly related to the total capacitance of the supercapacitor and can be used to qualitatively compare the capacitive behaviour of different samples^4^,^7^,^8^. As can be seen, the area of CV curve does not change much from Ni 0.5 M to Ni 1 M. However, it increases dramatically from Ni 1 M to Ni 2 M implying that the specific capacitance increases with the increase in the concentration of nickel. On further increasing the Ni content, the area of the CV curve drops quite significantly for the Ni 3 M sample. This increase from Ni 0.5 M to Ni 2 M may be attributed to the presence of more NiNPs in the system which increases the effective surface area for the redox reactions resulting in the higher pseudo-capacitance contribution to the overall capacitance of the electrode. On the contrary, the reduced area of the CV curve for Ni 3 M indicates that the specific capacitance decreases after the nickel nitrate concentration is increased above 2 M. This decrease in the specific capacitance is due to the increase in the particle size of NiNPs as seen in SEM which thereby results in a decrease in the overall specific surface area of the active material^27^. This indicates that the optimal concentration of NiNO_3_ to prepare NiNPs impregnated in carbonized wood is 2 M. To further confirm this and quantitatively determine the values of specific capacitance, EIS and galvanostatic charge-discharge measurements were conducted.

### Electrochemical Impedance Spectroscopy

**Figure 5:**
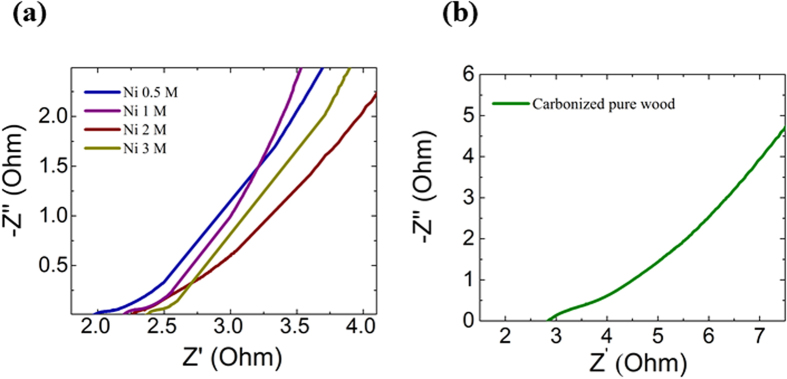
(**a**) Nyquist plots of Ni 0.5 M, Ni 1 M, Ni 2 M and Ni 3 M at frequency range from 10^6^ Hz to 0.1 Hz with potential amplitude of 0.1 V. (**b**) Nyquist plots of pure carbonized wood with same testing condition.

In order to utilize electrochemical supercapacitors as power storage devices, the electrodes should have a lower resistance (higher electrical conductivity) so as to achieve a lower power loss. The AC impedance technique, i.e. EIS measurements, was employed to quantitatively determine the equivalent series resistance (ESR) values for all the electrodes investigated in this study. The impedance measurements were carried out at a frequency range between 0.1 Hz and 10^6^ Hz. Figure 5 shows the Nyquist plots of Ni 0.5 M, Ni 1 M, Ni 2 M, Ni 3 M and pure carbonized wood electrodes under an applied potential amplitude of 0.1 V.

The Nyquist plots show a small arc in the high frequency region which is related to a charge transfer controlled process, and a straight line in the low frequency region corresponding to a diffusion controlled capacitive regime. The high frequency region where the curve first meets the x-axis gives the ESR value of the electrode. It was found that the ESR values of the NiNPs impregnated carbonized wood samples were 1.97 ohms, 2.19 ohms, 2.27 ohms and 2.37 ohms for Ni 0.5 M, 1 M, 2 M and 3 M respectively, which were less than the ESR value (2.90 Ohms) for the pure carbonized wood prepared under identical condition implying that the presence of NiNPs in the carbonized wood minimizes the power loss of the nickel/carbon nanocomposite based supercapacitors.

### Galvanostatic Charge-Discharge measurements

**Figure 6:**
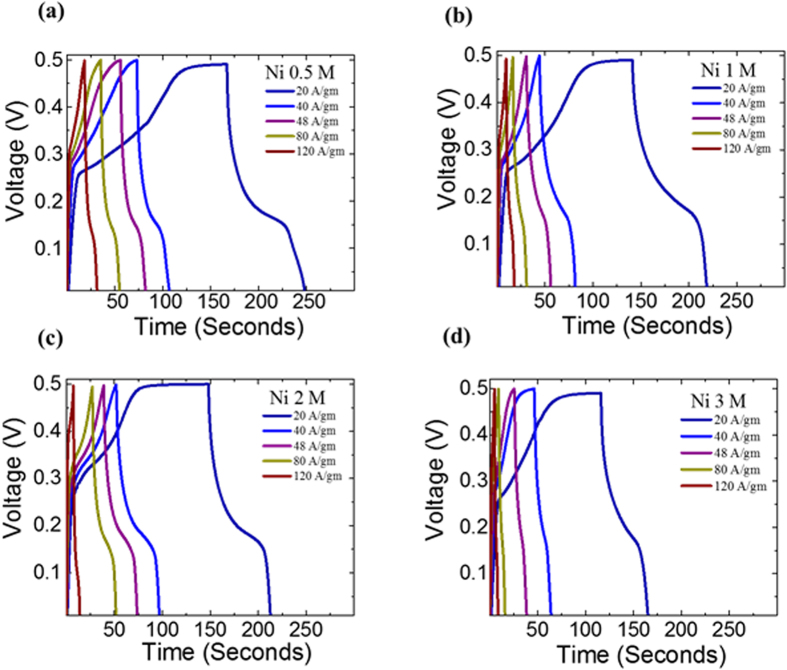
Galvanostatic charge-discharge curves for Ni 0.5 M, Ni 1 M, Ni 2 M and Ni 3 M at different current densities of 20 A/g, 40 A/g, 48 A/g, 80 A/g and 120 A/g. The data for 8 A/g is not shown in order to maintain the clarity in the figure.

In the next step, we conducted the galvanostatic charge-discharge experiments on the various NiNPs impregnated carbonized wood electrodes at current densities ranging from 8 A/g to 120 A/g over a potential window of 0.0–0.5 V^28^. Figure 6 shows the charge-discharge curves for NiNPs impregnated carbonized wood samples with various Ni concentrations. It was observed that the charging and discharging curves are non-linear with changes in slope being observed. This change in slope is due to the reaction between the electrolyte ions and the NiNPs impregnated carbonized wood electrode. Furthermore, the potential corresponding to the change in the slope matches with the peak potential of the CV curves, either anodic or cathodic peak, as seen in Fig. 4 and the non-linear charge discharge curve confirms that the NiNPs impregnated carbonized wood electrodes exhibit a psuedocapacitive behaviour.

The specific capacitance (F/g) of the NiNPs impregnated carbonized wood was calculated by using the discharge time and is given by Equation (1), where ‘i’ is the current, ‘m’ is the mass of the nanocomposite participating in the charge storage process, ‘Δt’ is the discharge time obtained from charge-discharge curves and ‘ΔV’ is the voltage window.


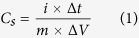


**Figure 7:**
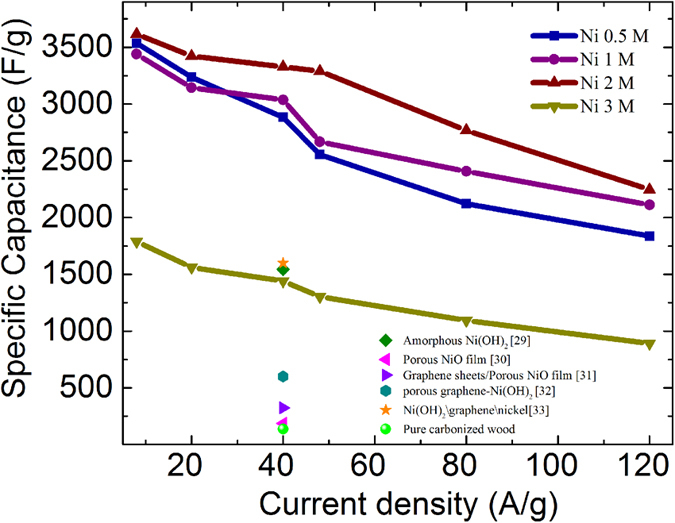
Specific capacitance for Ni 0.5 M, Ni 1 M, Ni 2 M and Ni 3 M at different current densities of (8 A/g–120 A/g) in 5 M KOH solution. Additionally, the specific capacitance of few common electrodes as reported in literature along with that of pure carbonized wood is also shown.

Figure 7 shows the specific capacitance (C_s_) values of all the NiNPs impregnated carbonized wood electrodes at various current densities. As can be observed, the C_s_ value decreases as current density increases. This is because, at high current densities the charging and the discharging process are limited only to the outer layers resulting in the decrease in number of accessible reaction sites in the electrode material. It is further observed that the C_s_ increases with the increase in the concentration of nickel content up to 2 M, but beyond which it decreases. Highest C_s_ value of about 3616 F/g is obtained for Ni 2 M sample at 8 A/g current density. Moreover, at 40 A/g Ni 2 M exhibits a C_s_ value of about 3328 F/g. This value is higher than the corresponding value reported in literature for electrodes made from amorphous Ni(OH)_2_ (~1544 F/g)^29^, porous NiO film (~188 F/g), graphene sheets/NiO film (~324 F/g)^30^, hierarchically porous graphene–Ni(OH)_2_ (hGN) hybrid hydrogel (~600 F/g)^31^, Ni(OH)_2_/graphene/Ni foam (~1600 F/g)^32^ Ni(OH)_2_/CNT/Nickel Foam (~3300 F/g)^33^ Ni(OH)_2_/Ni/graphene nanocomposite (~2609 F/g)^34^, and pure carbonized wood (136 F/g) tested under identical conditions. It is important to note that for being able to use a supercapacitor in fast charge-discharge applications it should show a high C_s_ even at higher current densities. Ni 2 M sample exhibits a very high C_s_ value of 2245 F/g at current densities as high as 120 A/g which offers it a great potential for use in applications that require ultra-high rate capabilities. In contrast to the Ni 2M, the Ni 3 M sample showed a lower C_s_ at all current densities as seen in Fig. 7. This is due to the presence of NiNPs which are very close to each other resulting in the aggregation of nanoparticles during the charge-discharge measurements. This aggregation causes a decrease in the total surface area and thereby reducing the accessibility of electrolyte towards the reactive sites.

The energy density (Wh/kg) and power density (W/kg) at various current densities for the NiNPs impregnated carbonized wood samples are calculated using equations 2 and 3 respectively.


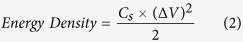



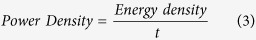


**Figure 8:**
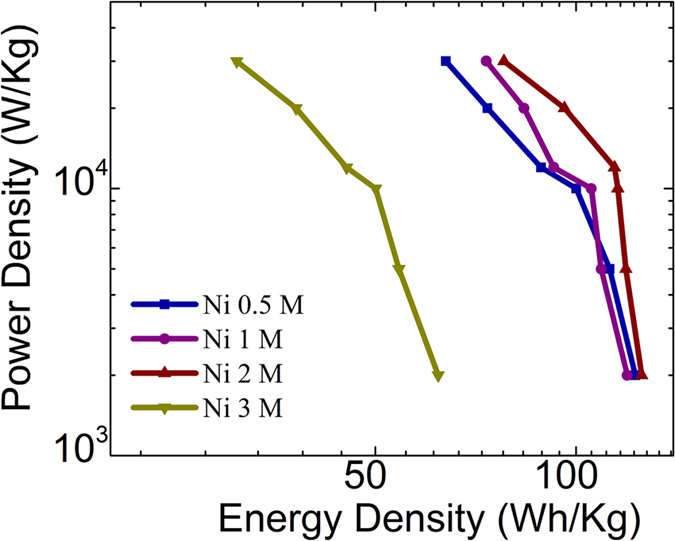
Ragone plot for Ni 0.5 M, Ni 1 M, Ni 2 M and Ni 3 M at various current densities.

Figure 8 shows the Ragone plot for the NiNPs impregnated carbonized wood samples at various current densities. From this figure, it can be seen that a highest power density of 30 kW/kg is obtained for Ni 2 M at 120 A/gm current density. And a highest energy density of 125 Wh/kg is obtained for Ni 2M sample at 8 A/gm current density. This clearly shows that the NiNPs impregnated carbonized wood electrodes possess an excellent combination of high energy storage and power density when compared to other materials presently available.

### Cyclic Life Test

**Figure 9:**
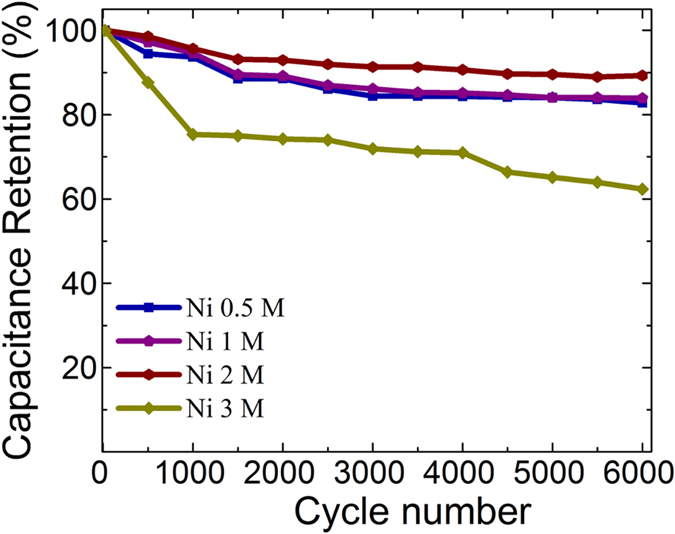
Cyclic life test data for Ni 0.5 M, Ni 1 M, Ni 2 M and Ni 3 M.

In order to investigate the performance of NiNPs impregnated carbonized wood electrodes in real-life applications, cyclic life test was performed wherein the galvanostatic charge-discharge experiments were repeated for 6000 cycles. Figure 9 represents the cyclic life tests of various NiNPs impregnated carbonized wood samples. It was observed that Ni 0.5 M, Ni 1 M, and Ni 2 M samples undergo a very slow decay in their specific capacitance up to 6000 cycles. These samples show a retention of more than ~83% after 6000 cycles of charge-discharge similar to the other composite electrodes^35^,^36^,^37^,^38^,^39^,^40^. In contrast to these samples, it was seen that Ni 3 M retains a capacitance of 62% of its initial capacitance after 6000 cycles. This relatively poor stability of Ni 3 M can be due to the aggregation of NiNPs during the continuous charging-discharging cycles which reduces the effective surface area thereby lowering its life time. Despite this, its specific capacitance after 6000 cycles is still higher than that of pure carbonized wood.

## Summary

In summary, a facile and environmentally friendly method for fabricating Ni/C nanocomposite from wood has been developed. The concentration of NiNPs in the carbonized wood was varied to understand the effect of nickel concentration on the performance of supercapacitor electrodes. Electrochemical characterization measurements such as CV, EIS and galvanostatic charge discharge were performed. The results showed that amongst all the Ni/C nanocomposite electrodes, Ni 2 M exhibits the best capacitive performance (3616 F/g at 8 A/g) which is attributed to the combination of pseudocapacitance of NiNPs and good electrical conductivity of the carbonized wood. Besides a high capacitive performance, Ni 2 M exhibits an excellent capacitance retention after 6000 cycles of repeated charge-discharge measurements suggesting its exciting potential as electrode materials for next-generation environmentally friendly supercapacitors.

## Additional Information

**How to cite this article**: Yaddanapudi, H. S. *et al*. Facile preparation of nickel/carbonized wood nanocomposite for environmentally friendly supercapacitor electrodes. *Sci. Rep.* **6**, 33659; doi: 10.1038/srep33659 (2016).

## Supplementary Material

Supplementary Information
