# Supplementary material for: Facile preparation of nickel/carbonized wood nanocomposite for environmentally friendly supercapacitor electrodes
Source: Sci Rep. 2016 Sep 21;6:33659. doi: 10.1038/srep33659 (PMC5030479; doi:10.1038/srep33659)
Supplement: Supplementary Information [file srep33659-s1.pdf]

Supporting Information

Facile preparation of nickel/carbonized wood nanocomposite for environmentally friendly supercapacitor electrodes

*Haritha Sree Yaddanapudi, Kun Tian, Shiang Teng & Ashutosh Tiwari**

Nanostructured Materials Research Laboratory,
Department of Materials Science and Engineering,
University of Utah, Salt Lake City, Utah, 84112

Determination of the nickel content in NiNPs impregnated carbonized wood electrodes:

The NiNPs impregnated carbonized wood samples were initially cut into two halves of which one half was used in determining the amount of nickel and the other half was used for further testing. The initial weight of one of these halves of the sample was taken and is denoted as 'W_1'. It was then transferred into a box furnace and heated to 900° C in air for 4 hours to convert all the carbon present in the sample into CO_2 and the nickel to nickel oxide. The weight of the remaining powder was weighed and is denoted as 'W_2'. By using the values of 'W_1' and 'W_2' and the molecular weights of nickel (58.69 g/mol) and nickel oxide (74.69 g/mol) in the Equation S1, the amount of nickel content in the samples can be estimated.

$$\% Ni = \frac{58.69 \times W_2}{74.69 \times W_1} \tag{S1}$$

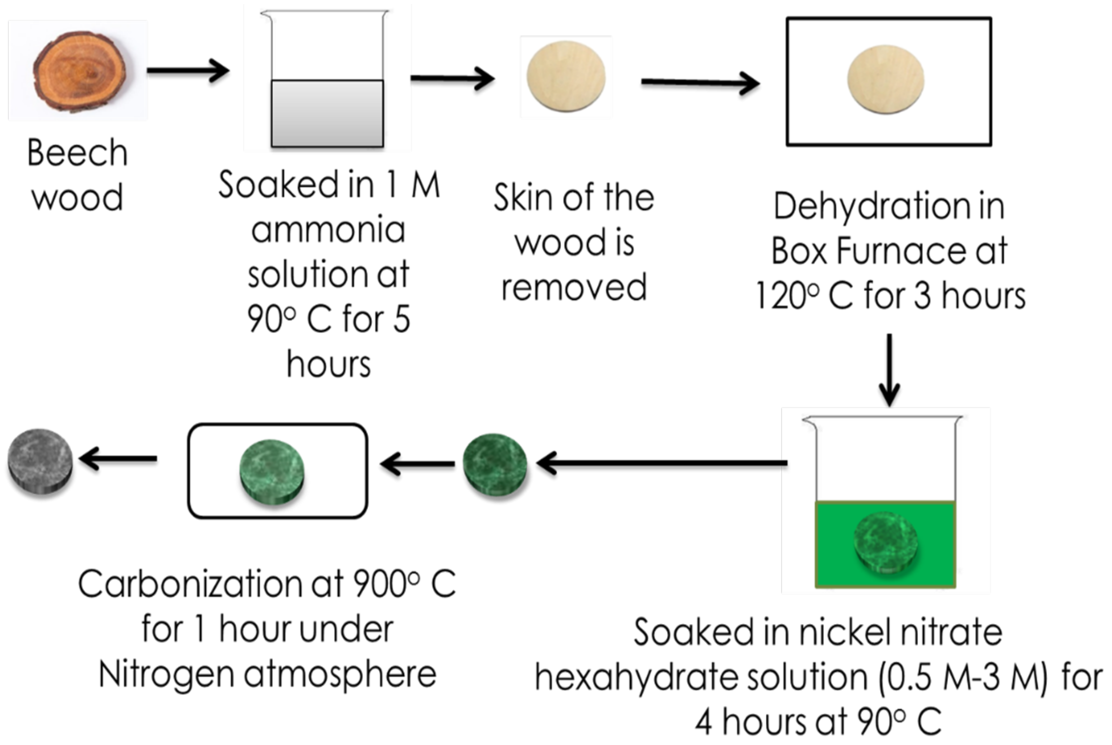

Figure S1. Schematic illustration of the procedure to prepare NiNPs impregnated carbonized wood electrodes.

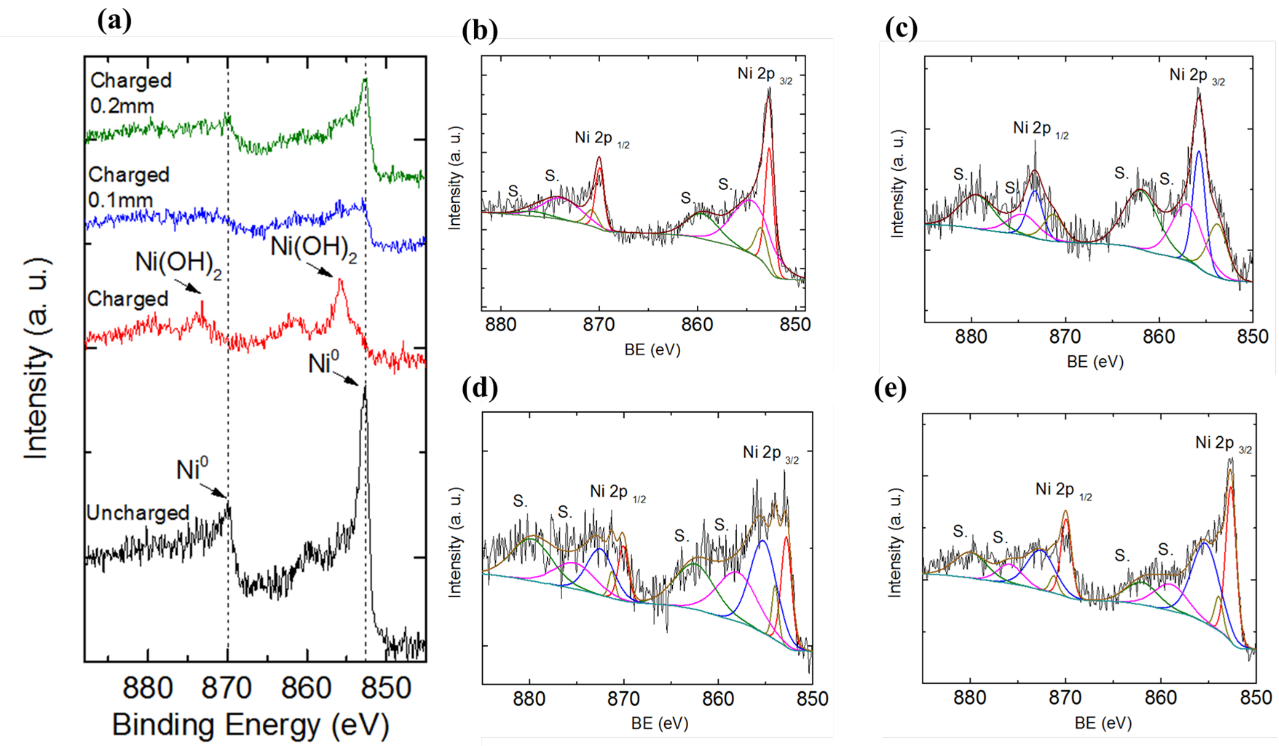

Figure S2. **(a)** High resolution XPS data showing Ni 2p peaks for the Ni 2M sample. Plots are shown for the uncharged sample, charged sample, sample obtained after mechanically etching 0.1 mm surface of the charged sample and the sample obtained after etching 0.2 mm of the uncharged sample. In all the cases, Ni $2p_{3/2}$ peak as well Ni $2p_{1/2}$ peak along with their satellite peaks (indexed as 'S') were observed. In the case of as-prepared uncharged sample **(b)**, Ni $2p_{3/2}$ and Ni $2p_{1/2}$ peaks were observed at the binding energy values of 852.6 eV and 869.9 eV, respectively and corresponds to the metallic phase of nickel (Ni^0) in the electrode. In contrast to the uncharged sample, the charged electrode showed a shift in the positions of Ni $2p_{3/2}$ and Ni $2p_{1/2}$ peaks towards the higher BE side **(c)**. Specifically, the Ni $2p_{3/2}$ and Ni $2p_{1/2}$ peaks were observed at the binding energy values of 855.8 eV and 873.2 eV, respectively. These peak positions correspond to 2+ oxidation state of Ni as observed in $Ni(OH)_2$. XPS spectrum of the sample obtained after etching 0.1 mm surface layer of the charged sample is shown in **(d)**. In this case the Ni $2p_{3/2}$ and Ni $2p_{1/2}$ peaks lie in between the corresponding positions for metallic nickel

(Ni^0) and Ni^{2+}. De-convolution of the peaks showed that the majority of the Ni was present in the form of $Ni(OH)_2$. On a contrary, when the charged sample was mechanically etched by 0.2 mm, the center of gravity of Ni $2p_{3/2}$ and Ni $2p_{1/2}$ peaks was found to be closer to Ni^0 **(e).** This implies that during the charging/discharging the redox reactions at the electrode are confined within a depth of 0.2 mm.

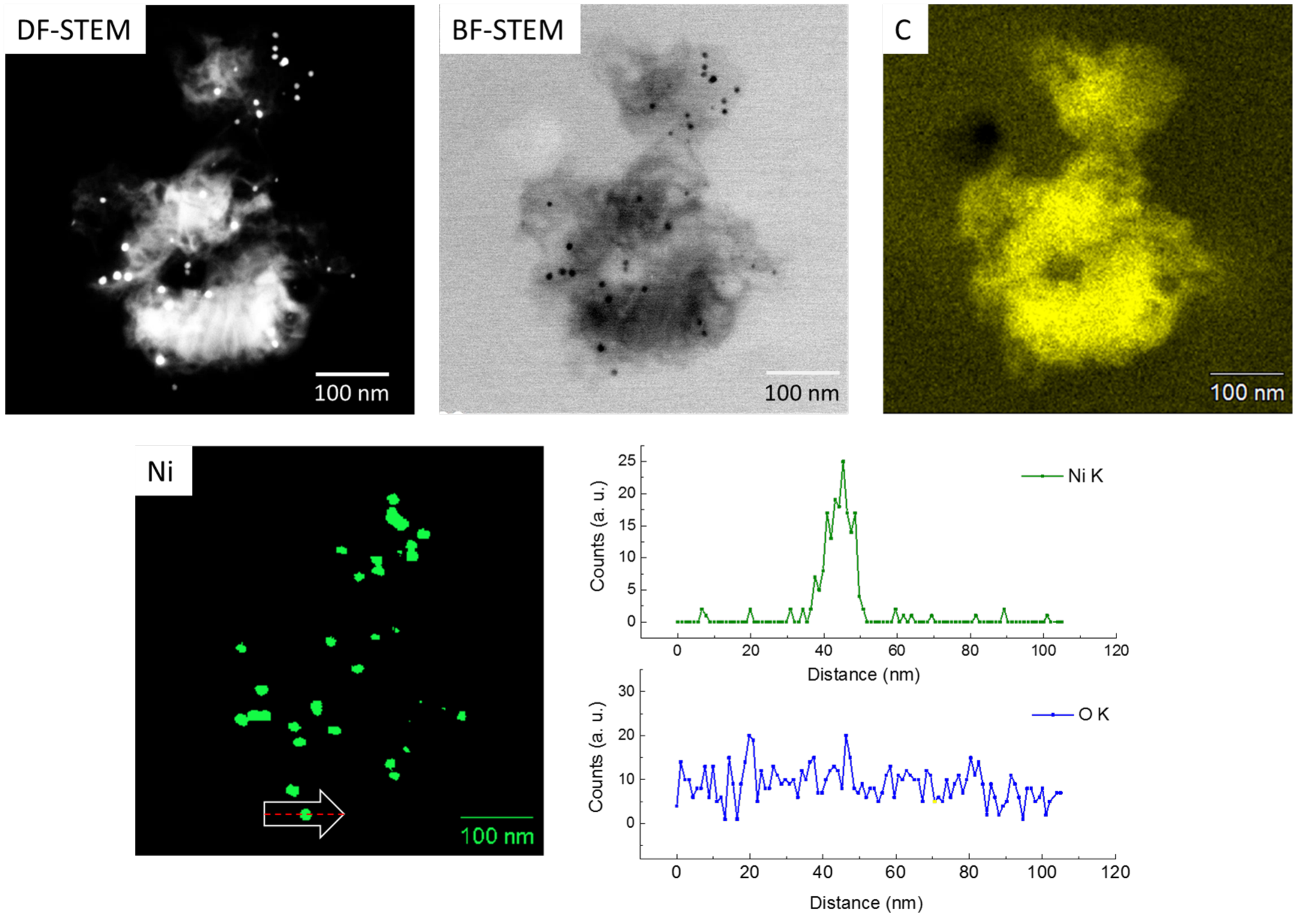

Figure S3. Scanning transmission electron microscopy (STEM) results for Ni 2M sample. Top left shows the DF-STEM image, top middle shows the BF- STEM image, top right shows the elemental distribution of carbon, bottom left shows the elemental distribution of nickel, and bottom right shows the EDX line profile across the nickel nanoparticle as indicated in the bottom left image.

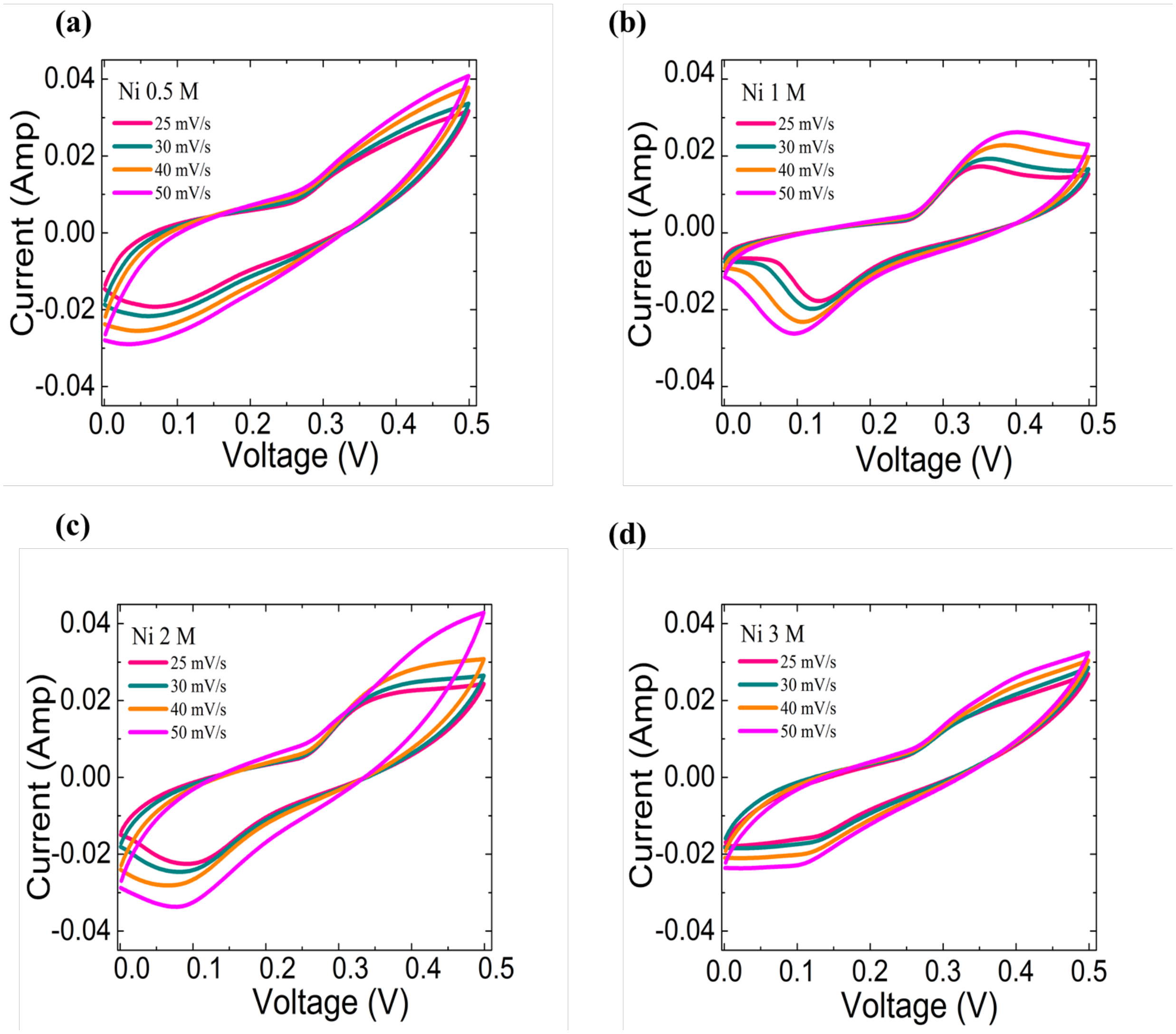

Figure S4. CV curves for Ni 0.5 M, Ni 1 M, Ni 2 M and Ni 3 M at high scan rates (varying from 25 mV/s to 50 mV/s).

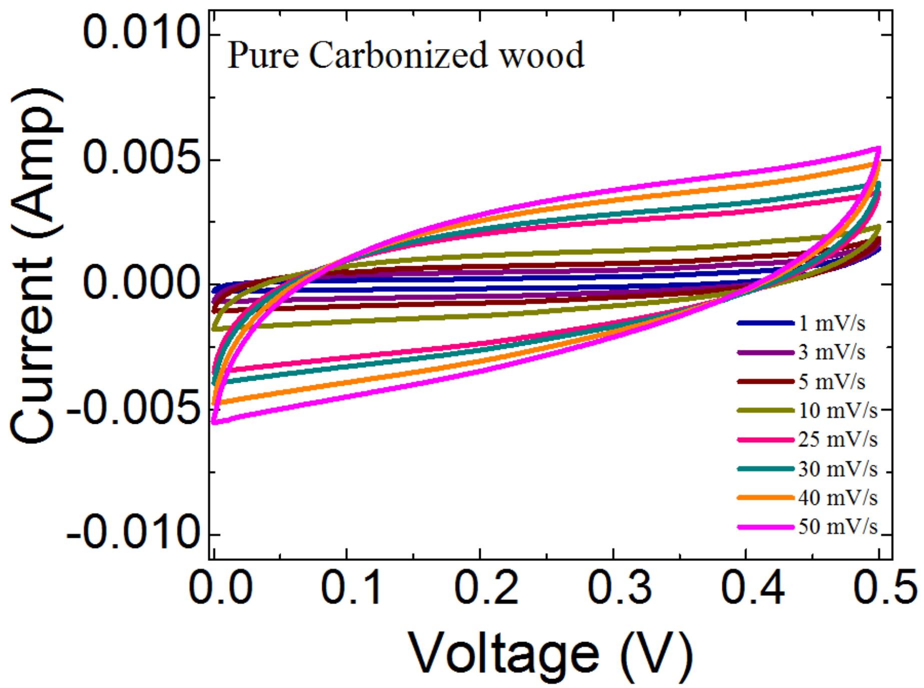

Figure S5. CV curves for pure carbonized wood at varying scan rates.

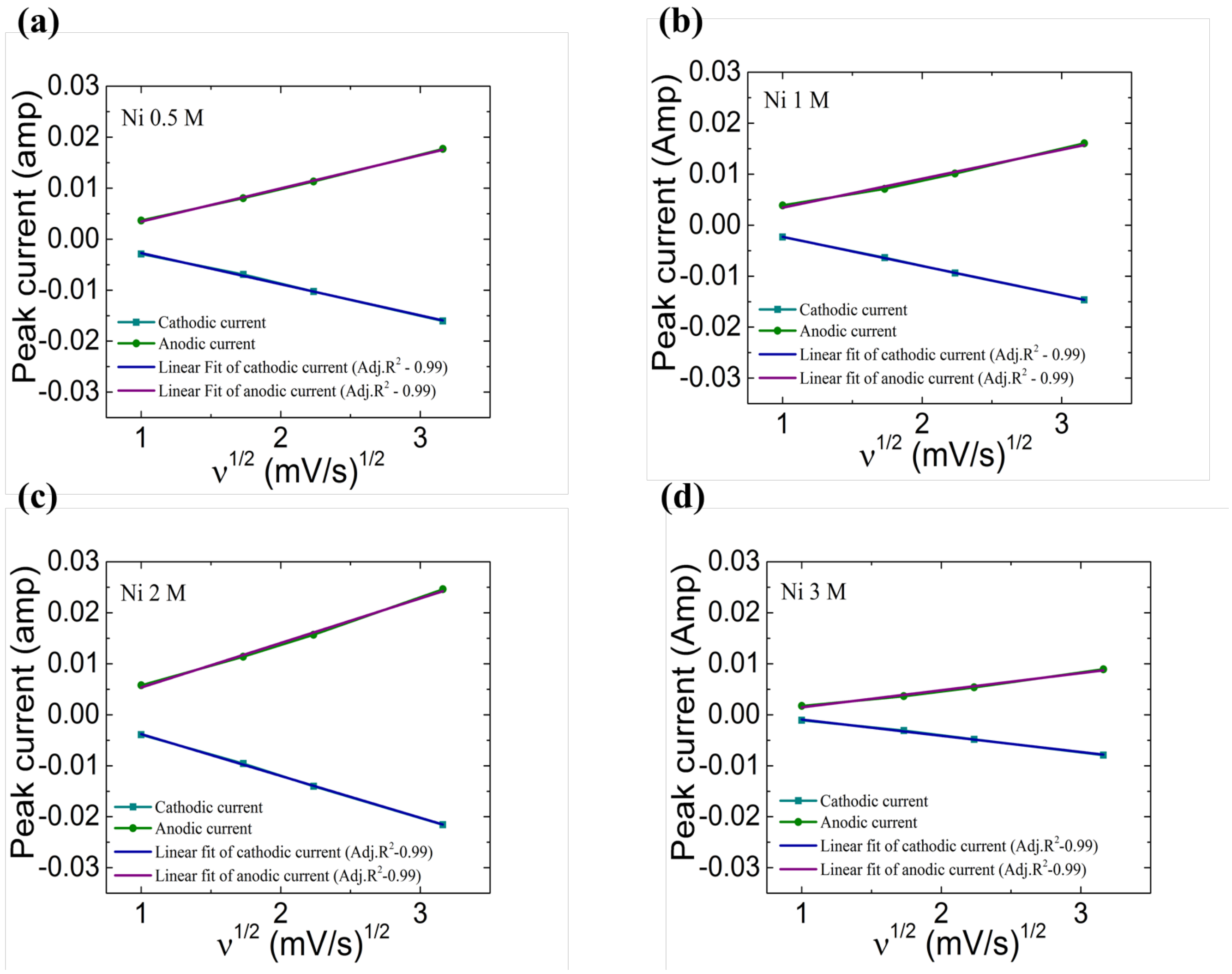

Figure S6. Plots showing linear relationship of the peak current with square root of scan rates indicating the process is diffusion controlled.

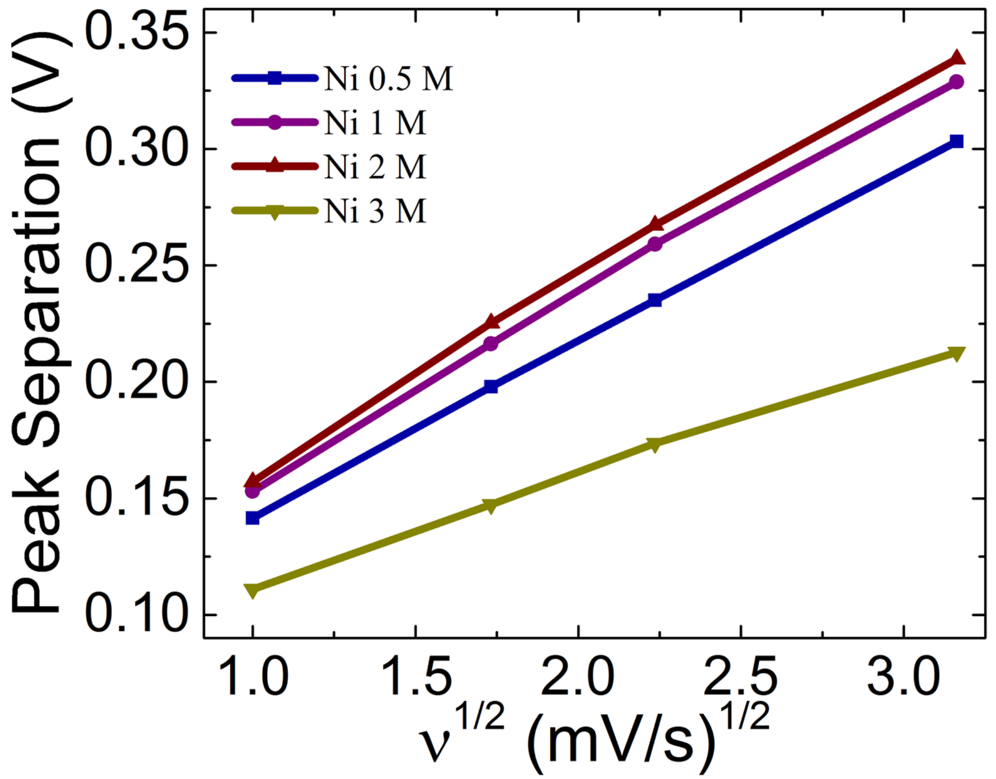

Figure S7. Plots showing peak separation vs square root of scan rates for various samples.
